# Quality Improvement: Evaluation of Team Communication Openness in Specialty and Emergency Veterinarians Who Regularly Use Communication Tools

**DOI:** 10.1111/vec.70134

**Published:** 2026-07-22

**Authors:** Richard H. Stone, Jennifer A. Adler, Melinda J. Larson, Lenore M. Bacek, Michael A. Rosen, Rochelle M. Low, Yea‐Jen Hsu, Albert W. Wu

**Affiliations:** ^1^ BluePearl Pet Hospital Tampa Florida USA; ^2^ Metropolitan Veterinary Associates Norristown Pennsylvania USA; ^3^ School of Medicine Johns Hopkins University Baltimore Maryland USA; ^4^ Mars Veterinary Health Vancouver Washington USA; ^5^ Bloomberg School of Public Health Johns Hopkins University Baltimore Maryland USA; ^6^ Armstrong Institute for Patient Safety and Quality, Johns Hopkins University Baltimore Maryland USA

**Keywords:** communication, communication openness, communication techniques, communication tools, patient safety

## Abstract

**Objective:**

To determine whether the regular use of structured communication tools is associated with greater communication openness among small animal specialty practitioners and emergency providers.

**Design:**

A cross‐sectional, anonymous, and voluntary survey of veterinary providers.

**Participants:**

Two hundred forty‐seven survey responses were received across three specialty veterinary college email listservs, including the American College of Veterinary Surgeons, the American College of Veterinary Internal Medicine, and the American College of Veterinary Emergency and Critical Care.

**Measurements and Main Results:**

Descriptive statistics were used to determine respondents’ awareness and use of communication tools, as well as perceptions of team communication openness. Most respondents (*n* = 201 [85.9%]) were unaware of Team Strategies and Tools to Enhance Performance and Patient Safety (TeamSTEPPS). Less than one‐third (*n* = 72 [30.8%]) reported that they or one of their colleagues had undergone formal training to enhance teamwork and communication in the hospital. Despite not being aware of specific TeamSTEPPS communication tools when referred to by name, many reported the use of communication tools when descriptions were provided. The call out, Situation–Background–Assessment–Response (or similar method), and handoff were the most commonly used communication tools. The debrief and huddle were the least frequently used at a regular interval. The check back and brief were variable in their frequency of use. Multivariable ordinal logistic regression analysis of communication tool utilization and communication openness found that frequent use of structured communication tools was associated with respondents reporting greater team communication openness.

**Conclusions:**

Awareness of specific TeamSTEPPS communication tools was low among specialty and emergency veterinarian respondents; however, many respondents reported consistent use of analogous structured communication techniques. Frequent use of structured communication tools was associated with communication openness.

**Abbreviations**
SBAR: Situation–Background–Assessment–Response
TeamSTEPPS: Team Strategies and Tools to Enhance Performance and Patient Safety

## 1 Introduction

Communication is critically important in hospital settings. In human health care, ineffective communication has been shown to be a significant cause of patient safety incidents and patient harm [1, 2]. Structured communication tools can help minimize such harm by facilitating the provision of safe, effective medical care [3]. Team Strategies and Tools to Enhance Performance and Patient Safety (TeamSTEPPS) is an evidence‐based group of communication and teamwork tools designed to optimize communication among healthcare providers, fostering a psychologically safe environment that encourages learning and ultimately improves performance of the healthcare team [4, 5].

TeamSTEPPS includes tools that facilitate transactions between individuals as well as in larger team settings [6]. With regular use, communication is improved by enhancing the exchange of information and supporting openness among team members [4]. This openness is characterized by team members’ comfort with speaking up when something unsafe is witnessed, ease in asking questions when something is perceived to be incorrect, and the receptivity of those in authority to such inquiries [7, 8]. The tools used for transfer of information and the presence of communication openness both contribute to overall team performance in healthcare settings [4, 9, 10].

In veterinary medicine, ineffective communication contributes to medical errors, as reported in reviews of claims records and patient safety events [11, 12, 13]. In one veterinary study, 80% of cases of alleged professional negligence were a result of communication error, and in 57% of cases, the delivery of care was influenced by poor communication, resulting in patient harm [14]. Similar to human medicine, the effectiveness of communication in veterinary settings can be affected by what information is exchanged, the structures used for communication, healthcare workers’ level of comfort speaking up to those in authority, and even their comfort level with asking colleagues for help [12]. However, little is known about the awareness and use of standardized communication tools in veterinary specialty and emergency medicine and how these tools influence communication openness.

We conducted a voluntary, anonymous survey of veterinary specialty and emergency medicine providers to determine the awareness and usage of various communication tools. We also sought to determine if the use of structured communication tools was associated with respondents’ perception of communication openness in their own veterinary hospital setting.

## 2 Materials and Methods

A voluntary, anonymous, cross‐sectional survey of veterinary specialty and emergency medicine providers was performed to determine their awareness and use of formal communication tools and whether these variables were associated with perceived communication openness. The survey was approved by the Johns Hopkins School of Public Health Institutional Review Board before distribution. Trialing of the survey was not performed.

An invitation to participate in the survey was sent to three specialty veterinary college email listservs: the American College of Veterinary Surgeons (via the Society of Veterinary Soft Tissue Surgery), the American College of Veterinary Internal Medicine, and the American College of Veterinary Emergency and Critical Care. The recruitment message, which included an embedded survey link, was unauthenticated so it could be forwarded to other respondents. The survey contained introductory language describing its purpose and how results would be used, as well as a statement of consent for participation.

The survey was live for 4 weeks from May 2, 2023, to June 2, 2023. All response data were collected via the InMoment survey platform using secure servers. Once opened, participants could terminate the survey at any time and skip any questions if they chose to do so. The 18‐item survey included questions to determine respondents’ characteristics, such as whether the respondent was patient facing, a hospital leader, gender, and length of time in practice (Appendix). Additional questions asked about practice size and whether cases were shared among services.

Respondents were asked if they were aware of TeamSTEPPS communication tools, including Situation–Background–Assessment–Response (SBAR), call out, check back, handoff, brief, huddle, and debrief. These communication tools were referred to by name, without definition, and respondents selected all that applied. Respondents were asked about the frequency with which they used specific communication tools in their practice location in a consistently structured manner. “Consistently structured” was defined as the team being aligned on a standardized method of using this communication tool. For these questions, the tools were not referred to by name but instead described using definitions (Table 1). Respondents were also asked whether they or their colleagues had undergone formal communication or teamwork training. Responses for use of communication tools were collected with Likert scale formatting.

**TABLE 1:** Definitions of TeamSTEPPS communication training tools.

Communication tools	Definition
**Transactional tools**	
SBAR	Tool for concisely communicating critical information regarding a patient's condition that requires immediate attention and action
Call out	Tool for communicating during an emergent situation that verbally informs and helps direct responsibility to those on the caregiving team
Check back	A system of closed‐loop communication where one team member (the sender) communicates patient information to a recipient; the recipient provides a response or feedback, and the original sender then verbally confirms that the message was received
Handoff	Tool for transfer of information (along with authority and responsibility) during transitions in care within the hospital that includes an opportunity to ask questions, clarify, and confirm pertinent patient information
**Team event tools**	
Brief	Host a short team session before the beginning of a day/shift, procedure, or intervention to share the plan, assign team member roles and responsibilities, establish expectations, and anticipate outcomes and likely contingencies
Huddle	Host an ad hoc team meeting to reestablish situational awareness related to patient care, reinforce plans already in place for the delivery of care, and assess the need to adjust the plan
Debrief	Host an informal team session after an event or procedure, designed to improve team performance and effectiveness through the review of lessons learned and reinforcement of positive behaviors

*Note*: Transactional TeamSTEPPS tools facilitate information exchange between individuals. Team event TeamSTEPPS tools are used by larger groups to coordinate patient care activities.

Abbreviations: SBAR, Situation–Background–Assessment–Recommendation; TeamSTEPPS, Team Strategies and Tools to Enhance Performance and Patient Safety.

Communication openness survey questions were adapted from version 2.0 of the Agency for Healthcare Research and Quality Survey on Patient Safety Culture [7], which is used to determine the extent to which a hospital culture supports patient safety [7, 15]. These responses were also collected using Likert scale formatting.

The survey was directed toward veterinary healthcare providers. Specialty college email listservs were used to narrow the participant population to veterinarians, technicians, and other patient‐facing practitioners working in specialty and emergency hospitals. After completion of data collection, only patient‐facing veterinarians were included due to low responses among other roles. Respondents were excluded if they failed to complete the survey. However, “I prefer not to answer” was an accepted response to some individual demographic questions, and “not applicable” was an accepted response to the individual communication openness questions (Appendix). Respondents that were not patient facing were excluded.

### 2.1 Statistical Methods

Respondents’ characteristics, their use of specific communication tools, and responses related to team communication openness were described using frequencies and percentages. Ordinal logistic regression was used to compare the frequency of tool use between specialties. Following the guidelines in the Survey on Patient Safety Culture analysis manual, a composite communication openness score was generated for each respondent based on the number of top box responses among the four included items [7, 8]. Based on manual guidance, the top box responses for positively phrased items were “Most of the Time” and “Always,” and for negatively phrased items, the top box responses were “Never” and “Rarely ” [7]. The composite communication openness score for each respondent ranged from 0 to 4, with 4 the highest score possible.

To assess the frequency of communication tool use and the relationship with communication openness, two aggregate measures were created: (1) the number of transactional communication tools (e.g., SBAR, call out, check back, and handoff) used at least several times a week and (2) the number of team event tools (e.g., brief, huddle, and debrief) used at least several times a week. Multivariable ordinal logistic regression was performed to examine the association between the use of these tools and perceived communication openness. Models were adjusted for respondents’ characteristics, including gender, practice experience (<5, 5–14, or ≥15 years), leadership role (yes/no), department (e.g., internal medicine, emergency and critical care, other), and whether respondents shared patients across services (yes/no).

Statistical significance of the frequency of tool use (i.e., whether respondents used the tools at least several times a week) was assessed with *χ* ^2^ tests when individuals who were aware of the listed communication tools were compared with those who were not.

## 3 Results

Over the 4‐week period, 247 responses were received. The combined email lists had approximately 4321 total subscribers at the time of the survey (American College of Veterinary Emergency and Critical Care, *n* = ∼900; American College of Veterinary Internal Medicine, *n* = ∼3021; Society of Veterinary Soft Tissue Surgeons, *n* = ∼400), translating to a ∼5.7% response rate. The survey took less than 6 min to complete (mean: 5.8 min, range: 2.3–19.5). Technicians (*n* = 8) were excluded from analysis due to the small number of responses. Respondents who did not answer questions related to communication openness, awareness, and use of formal communication tools, as well as individual and practice characteristics, were also excluded (*n* = 2). In addition, respondents who were not in patient‐facing roles were excluded (*n* = 3). A total of 234 individuals were included in the final analytic sample.

Most respondents were women (*n* = 183 [78.2%]); 45 (19.2%) respondents were men, and six (2.7%) self‐described as other or preferred not to say. Half (*n* = 118 [50.2%]) the respondents had been in practice less than 5 years, 101 (43.2%) from 5 to 14 years, and 15 (6.4%) had been in practice for 15 years or more. Most respondents (*n* = 143 [61.1%]) did not hold a leadership role in their hospital.

Most respondents were emergency and critical care clinicians (*n* = 109 [46.6%]) and internal medicine clinicians (*n* = 65 [27.8%]). The remainder were from other services (*n* = 60 [25.4%]), including surgery, neurology, oncology, cardiology, and other (Table 2). Most respondents indicated they practiced in a multidisciplinary environment (*n* = 228 [97.4%]), with many sharing cases across services (*n* = 205 [87.6%]).

**TABLE 2:** Characteristics of 234 specialist and emergency veterinarians participating in a survey about communication tool awareness and use.

	*n* = 234	Percentage (%)
Gender		
Female	183	78.2
Male	45	19.1
Other	6	2.6
Years in practice		
<5 years	118	50.4
5–14 years	101	43.2
≥15 years	15	6.4
Leadership role		
Yes	91	38.9
No	143	61.1
Department		
Internal medicine	64	27.3
Emergency and critical care	110	47
Neurology	24	10.2
Surgery	24	10.2
Other	12	5.1
Patients shared across services		
Yes	205	87.6
No	29	12.4

Respondents’ awareness of communication tool names varied (Table 3). They were most aware of debrief (*n* = 138 [59%]), huddle (*n* = 122 [52.1%]), and handoff (*n* = 114 [49.7%]), and least aware of check back (*n* = 37 [15.8%]), SBAR (*n* = 37 [15.8%]), and call out (*n* = 32 [13.7%]). Most respondents (*n* = 201 [85.9%]) were unaware of the TeamSTEPPS framework. Less than one‐third (*n* = 72 [30.8%]) reported that they or one of their colleagues had undergone formal training to enhance teamwork and communication in the hospital, while the remainder had not (*n* = 132 [56.4%]) or were not sure (*n* = 30 [12.8%]).

**TABLE 3:** Awareness of TeamSTEPPS communication tools among specialty and emergency veterinarian survey respondents when referred to by name (*n* = 234).

Communication tool	Number of respondents aware	Percentage (%)
Debrief	138	59.0
Huddle	122	52.1
Handoff	114	48.7
Brief	65	27.8
Check back	37	15.8
SBAR	37	15.8
Call out	32	13.7

Abbreviations: SBAR, Situation–Background–Assessment–Recommendation; TeamSTEPPS, Team Strategies and Tools to Enhance Performance and Patient Safety.

Based on tool description, the call out, SBAR (or similar method), and handoff were the most commonly used communication techniques, with 72.6%, 68.4%, and 77.4% of respondents, respectively, reporting daily use or use several times per week (Table 4). The least frequently used techniques were the debrief (6.8%) and huddle (10.7%), both used daily or several times per week. The check back and brief were mixed in their frequency of use (Table 4). Further examination of use across three major specialties (i.e., internal medicine, emergency and critical care, surgery) suggested that respondents in the emergency and critical care specialty used SBAR and debrief more frequently compared with respondents in internal medicine. Additionally, brief was used more by respondents from the surgery specialty than respondents from the other two specialties (Tables 5 and 6). No differences were found with the use of other tools.

**TABLE 4:** Reported use of TeamSTEPPS communication tools among specialty and emergency veterinarian survey respondents based on description of each tool (*n* = 234).

	Frequency of use		
Communication tool	Daily or several times a week *n* (%)	Weekly *n* (%)	Monthly or less, or never *n* (%)
Call out	170 (72.6)	27 (11.5)	37 (15.8)
SBAR	160 (68.4)	26 (11.1)	48 (20.5)
Check back	111 (47.4)	35 (15.0)	88 (37.6)
Handoff	181 (77.4)	20 (8.5)	33 (14.1)
Brief	109 (46.5)	27 (11.5)	98 (41.9)
Huddle	25 (10.7)	25 (10.7)	174 (74.4)
Debrief	16 (6.8)	20 (8.5)	198 (84.6)

Abbreviations: SBAR, Situation–Background–Assessment–Recommendation; TeamSTEPPS, Team Strategies and Tools to Enhance Performance and Patient Safety.

**TABLE 5:** Survey respondent‐reported use of TeamSTEPPS communication tools across 234 IM, ECC, and surgery specialty veterinarians based on description of each communication tool.

	SBAR, *n* (%)			Brief, *n* (%)			Debrief, *n* (%)		
Frequency of use by service	IM	ECC	Surgery	IM	ECC	Surgery	IM	ECC	Surgery
Daily or several times a week	39 (60.0)	87 (79.8)	18 (72.0)	33 (50.8)	35 (32.1)	21 (84.0)	2 (3.1)	8 (7.3)	2 (8.0)
Weekly	9 (13.8)	6 (5.5)	5 (20.0)	5 (7.7)	16 (14.7)	1 (4.0)	3 (4.6)	12 (11.0)	3 (12.0)
Monthly or less, or never	17 (26.2)	16 (14.7)	2 (8.0)	27 (41.5)	58 (53.2)	3 (12.0)	60 (92.3)	89 (81.7)	20 (80.0)

Abbreviations: ECC, emergency and critical care; IM, internal medicine; SBAR, Situation–Background–Assessment–Recommendation; TeamSTEPPS, Team Strategies and Tools to Enhance Performance and Patient Safety.

**TABLE 6:** Ordinal logistic regression results for comparison of the use of TeamSTEPPS communication tools across veterinary specialties, including ECC, IM, and surgery, as reported by 234 specialist and emergency veterinarians.

Tool	Comparison	OR	95% CI	*p* value
SBAR	ECC vs. IM	2.75	1.33–5.67	0.006
Brief	Surgery vs. IM	5.26	1.61–17.21	0.006
Brief	Surgery vs. ECC	2.15	1.00–3.30	<0.002
Debrief	ECC vs. IM	2.92	0.99–8.60	0.052

*Note*: The regression models adjusted for gender, years in practice, leadership role, and sharing patients across services. Only statistically significant differences are presented.

Abbreviations: CI, confidence interval; ECC, emergency and critical care; IM, internal medicine; OR, odds ratio; SBAR, Situation–Background–Assessment–Recommendation; TeamSTEPPS, Team Strategies and Tools to Enhance Performance and Patient Safety.

Respondents gave generally positive assessments of team communication openness (Table 7). Most indicated that staff speak up when they see something that may negatively affect patient care (86.3% choosing “most of the time” or “always”), and 73.1% reported that staff are not afraid to ask questions when something seems wrong. Although 80.2% reported that those in authority are open to safety concerns, only 53.5% reported that staff speak up when someone with greater authority does something unsafe. The possible range of the communication openness composite score was between 0 and 4, as it is calculated as the number of communication openness survey items with a top box response. The distribution showed that 4.7% (*n* = 11) of respondents had a composite score of 0, indicating that none of the questions were answered in alignment with observed communication openness, 9.8% (*n* = 23) scored a 1, 13.7% (*n* = 32) a 2, 32.9% (*n* = 77) a 3, and 38.9% (*n* = 91) a 4, representing complete alignment across all four questions.

**TABLE 7:** Team communication openness reported by 234 specialty and emergency veterinarians using a communication openness scale following the guidelines in the Survey on Patient Safety Culture analysis manual [13].

Communication openness scale	1 = *Never* *n* (%)	2 = *Rarely* *n* (%)	3 = *Sometimes* *n* (%)	4 = *Most of the time* *n* (%)	5 = *Always* *n* (%)
In my service/department, staff speak up if they see something that may negatively affect patient care	2 (0.9)	3 (1.3)	27 (11.5)	125 (53.4)	77 (32.9)
When staff in my service/department see someone with more authority doing something unsafe for patients, they speak up	9 (3.9)	24 (10.4)	74 (32.2)	102 (44.4)	21 (9.1)
When staff in my service/department speak up, those with more authority are open to their patient safety concerns	1 (0.4)	7 (3.0)	38 (16.4)	107 (46.1)	79 (34.1)
In my service/department, staff are afraid to ask questions when something does not seem right (reverse coded)	32 (13.7)	139 (59.4)	53 (22.7)	8 (3.4)	2 (0.9)

Frequent use of structured transactional communication tools and team event tools was associated with greater reported communication openness (Table 8). Specifically, respondents who used more structured transactional communication tools at least several times a week yielded higher communication openness composite scores (odds ratio: 1.61, 95% confidence interval: 1.33–1.95; *p* < 0.001) after adjusting for other covariates. Similarly, use of more team event tools, occurring at least several times a week, was also associated with higher communication openness composite scores (odds ratio: 1.45, 95% confidence interval: 1.02–2.06; *p* = 0.036).

**TABLE 8:** Regression analysis of association between reported use of communication tool(s) at least several times weekly and communication openness scores, according to 234 specialty and emergency veterinarians.

	OR	95% CI	*p* value
Number of transactional communication tools used at least several times a week	**1.61**	1.33–1.95	**<0.001**
Number of team event tools used at least several times a week	**1.45**	1.02–2.06	**0.036**
Gender			
Female	1		
Male	1.53	0.81–2.91	0.189
Other	0.81	0.17–3.85	0.789
Years in practice			
<5 years	1		
5–14 years	1.02	0.61–1.70	0.948
≥15 years	2.57	0.88–7.46	0.083
Department			
Internal medicine	1		
Emergency and critical care	0.82	0.46–1.49	0.518
Other	1.01	0.51–1.99	0.974
Share patients across services			
No	1		
Yes	0.83	0.40–1.72	0.618

*Note*: Ordinal logistic regression models were conducted. The association between use of more communication tools and communication openness was not significantly related to demographic factors, department, or sharing of cases across services. Statistically significant results are reported in bold.

Abbreviations: CI, confidence interval; OR, odds ratio.

## 4 Discussion

The current study describes the awareness and use of formal communication tools among specialty and emergency veterinarians and the association between the use of these tools and team communication openness.

When referred to by their names, awareness of specific communication tools was variable but generally low. The greatest awareness was of the debrief, huddle, and handoff tools, terms used commonly outside of medical team communication, which explains their higher rates of familiarity. Respondents were least aware of the check back, SBAR, and call out tools, terms that, in the authors’ experience, are used less commonly outside of communication training and healthcare contexts.

Formal communication tool familiarity, or lack thereof, did not consistently correspond with actual communication tool use. For example, while respondents had the highest awareness of the terms huddle and debrief, the level of awareness of these terms did not correlate with the reported use of the communication tool when asked in context of the definition. Conversely, many respondents reported regular use of several of the communication tools despite not knowing them by name—notably, call out, hand off, or an SBAR‐like technique. Although formal awareness of these names was low, it is interesting to note that more than two‐thirds of respondents reported using one of these tools daily to several times a week. Structured training among veterinary teams could further enhance the use and effectiveness of these tools.

The tools mentioned in the current study were categorized as transactional or team event tools, as described in the TeamSTEPPS framework [6]. The transactional tools, such as SBAR, call out, check back, and handoff, involve two or more people exchanging information to achieve a shared understanding [16]. In contrast, team event tools, such as the brief, huddle, and debrief, generally involve meetings of larger groups and are intended to facilitate the coordination of essential activities related to patient care [4]. Results showed that transactional communication tools were more commonly used than team event tools. A potential reason for this discrepancy in use is that team events generally require greater investment from hospital leadership and team participation in terms of time and resource allocation. In contrast, transactional tools can be spearheaded by individuals or departments.

Our findings demonstrate differences in how specialty services use tools of communication. Respondents in the emergency and critical care specialty were more likely to report the use of SBAR and debrief. It is possible that the nature of critical care medicine may necessitate more frequent use of debriefs, specifically after patient arrest and CPR events. In comparison, respondents in the surgery specialty reported use of the brief more frequently compared with their counterparts in other specialties. Briefs are particularly useful for planning before a shift or procedure; thus, surgery teams may benefit from more frequent coordination and planning due to the nature of the service. These variations in communication practices underscore the importance of training and support that is tailored to meet the specific needs of each specialty.

Despite generally positive responses about communication openness, our results suggest there is still hesitation to speak up when authority figures are involved in unsafe acts, with 46.5% of respondents reporting staff do not speak up when someone with higher authority is taking unsafe actions. This is consistent with findings in human health care and other safety‐critical organizations [17, 18, 19]. Our results showed that the hesitancy to speak up could be underrepresenting the entire veterinary team's hesitancy, as respondents included in this analysis are clinicians. The magnitude of the power dynamic experienced by technicians and other team members may not be fully realized, nor may clinicians be aware of every instance in which a team member has a concern [20]. This is noteworthy for hospital and department leaders, as the failure to speak up does not necessarily indicate team members’ lack of concerns related to patient safety or delivery of care. In fact, this finding illustrates the need for hospital leaders to intentionally provide venues and methods for team members to voice their concerns without fear of retribution.

Frequent use of any of the communication tools was associated with higher communication openness scores. Notably, higher levels of communication openness were reported when more TeamSTEPPS tools were used at least several times weekly. Specialty and emergency veterinary care often involves managing complex, high‐risk patients. In the authors’ experience, the key to continuous improvement and provision of safe care in high‐risk settings is the comfort with openly discussing risk and safety events. Similar to human health care, specialty and emergency veterinary care teams benefit from activities that enhance communication openness and, subsequently, a culture of safety.

The current study was not designed to quantify how communication openness affects safety culture and patient outcomes, but studies in human health care have demonstrated such benefits. For example, communication openness has been shown to have a positive effect on reporting of nosocomial infections and team cohesion [21]. In another study of hospital nurses, psychological safety and job satisfaction decreased turnover, improved patient safety, and were enhanced by open communication [22]. Our study was likewise not constructed to describe correlations between communication openness and technician turnover rates, but similar pressures do exist in the veterinary profession that warrant further study.

Awareness of TeamSTEPPS communication training was low in the specialty and emergency veterinarians who participated in our survey. Fewer than one‐third of respondents reported they or their colleagues had undergone any formal communication training, TeamSTEPPS or otherwise, which is frequently undertaken in human health care with the aim of improving teams’ nontechnical skills, in turn improving patient experience and safety [23, 24, 25, 26, 27, 28, 29]. We predict that greater awareness and use of available teamwork and communication training programs could similarly benefit veterinary teams and their patients through enhanced communication and culture of safety.

The current study is based on the findings of a voluntarily completed survey, and the results may not apply to specialty and emergency veterinarians who did not participate. Our response rate is estimated by assuming all subscribers of the included listservs received the survey, but this could not be confirmed. In addition, we could not quantify the number of veterinary providers who may have received a forwarded survey. Designing the survey so that recipients could forward it to colleagues could have theoretically allowed for repeated participation; however, no duplicate submissions were detected upon comparison of individual responses.

A portion of the survey was adapted from the Agency for Healthcare Research and Quality Survey on Patient Safety Culture 2.0 [7]. We acknowledge that although there are similarities between human and veterinary healthcare teams and environments, the survey tool was validated in human health care and not veterinary settings.

Our results may be biased by a higher response rate from respondents with an interest in communication. Our respondent group also does not fully represent specialty and emergency veterinary medicine, as few veterinary technician responses were received and thus were not included in the analysis. Veterinary teams are composed of clinicians, technicians, customer service associates, and other team members, with power dynamics experienced differently due to role diversity. Thus, the full extent of communication openness may not have been captured in our results. Additionally, our respondent population was predominantly sourced from multidisciplinary hospital environments.

As an area of future study, a closer look at the use of communication tools and openness across the full spectrum of veterinary team roles and practice types is warranted. We recognize that it is possible that individuals with greater communication openness may be more inclined to use communication techniques on a regular basis; thus, we were unable to establish directionality. However, it is noteworthy that an intended outcome of training and use of these techniques is an enhancement of communication openness. This study is the first of its kind in veterinary medicine, determining awareness of structured communication tools, their use by a subset of specialty and emergency veterinarians, and their association with team communication openness. These findings will act as a baseline for future comparisons in veterinary hospital settings when the effects of implementing communication tools are assessed.

## Author Contributions

**Richard H. Stone**: conceptualization, investigation, writing – original draft, methodology, writing – review and editing, supervision, formal analysis, project administration, visualization, resources. **Jennifer A. Adler**: conceptualization, writing – review and editing, formal analysis, visualization. **Melinda J. Larson**: writing – review and editing. **Lenore M. Bacek**: writing – review and editing. **Michael A. Rosen**: conceptualization, writing – review and editing, validation. **Rochelle M. Low**: conceptualization, writing – review and editing. **Yea‐Jen Hsu**: conceptualization, software, formal analysis, writing – review and editing, data curation. **Albert W. Wu**: conceptualization, writing – review and editing, validation.

## Ethics Statement

This survey study protocol was approved by the Johns Hopkins School of Public Health Institutional Review Board.

## Conflicts of Interest

The authors declare no conflicts of interest.

## Data Availability

The data that support the findings of this study are available from the corresponding author upon reasonable request.

## 1 Communication and Teamwork in Vet Med Survey

Do you work in a patient‐facing role within your hospital?

Yes

No

Are you in a hospital leadership role?

Yes

No

*[If Q1 = Yes]* Which of the following titles best describes your patient‐facing role? *[Randomize choices]*

Veterinarian

Veterinary Technician

Client Services

Other (specify):

*[If Q3 = Veterinarian or Veterinary Technician]* Which of the following best describes your service/department?

Cardiology

Dermatology

Emergency and Critical Care

Exotics

Internal Medicine

Neurology

Nutrition

Oncology

Ophthalmology

Radiology

Rehabilitation

Surgery

Other (specify):

How many total services (e.g., internal medicine, emergency, surgery, etc.) currently exist within your hospital?

1

2–4

5–7

8–10

More than 10

Not applicable

Is your hospital typically staffed with overnight medical personnel?

Yes

No

*[If Q5<> Not applicable]* Do you share patients between individual caregivers within the same service at your hospital? For example, is case responsibility transferred or shared between clinicians?

Yes

No

*[If Q5 = 2 or more AND <> Not applicable]* Do you share patients across services within your hospital? In other words, is case responsibility transferred or shared between different services?

Yes

No

For the next set of questions, please consider the teamwork and information exchange at your hospital. Using a scale where 5 = *Strongly agree* and 1 = *Strongly disagree*, please rate your level of agreement with each statement. If you are unsure or a statement does not apply, please select N/A. *[Randomize choices]*

**Table:**

	1 = *Strongly disagree*	2 = *Disagree*	3 = *Neither agree or disagree*	4 = *Agree*	5 = *Strongly agree*	N/A
In my service/department, we work together as an effective team	O	O	O	O	O	O
During busy times, staff in my service/department help each other	O	O	O	O	O	O
There is a problem with disrespectful behavior by those working in my service/department	O	O	O	O	O	O
*[If Q7* = *Yes]* When transferring patients between clinicians within the same service/department, important information is often left out	O	O	O	O	O	O
*[If Q8* = *Yes]* When transferring patients from one service/department to another, important information is often left out	O	O	O	O	O	O
During shift changes, important patient care information is often left out	O	O	O	O	O	O
During shift changes, there is adequate time to exchange all key patient care information	O	O	O	O	O	O

Describe the frequency with which you utilize each of the following communication techniques in a consistently structured manner in your service or hospital. Note, “consistently structured” in this question means your team has aligned on a predictable, shared method of this communication type. *[Randomize choices]*

**Table:**

	*Never*	*Monthly or less often*	*Weekly*	*Several times per week*	*At least daily*
Technique for concisely communicating critical information on a patient's condition that requires immediate attention and action	O	O	O	O	O
Technique for communicating during an emergent situation that verbally informs and helps direct responsibility to those on the caregiving team	O	O	O	O	O
A system of closed loop communication where one team member (the sender) communicates patient information to a recipient, the recipient provides a response or feedback, and the original sender then verbally confirms that the message was received	O	O	O	O	O
Technique for transfer of information (along with authority and responsibility) during transitions in care within the hospital that includes an opportunity to ask questions, clarify, and confirm pertinent patient information	O	O	O	O	O

Describe the frequency with which your service (within your own hospital) utilizes the following team communication events. *[Randomize choices]*

**Table:**

	*Never*	*Monthly or less often*	*Weekly*	*Several times per week*	*At least daily*
Host a short team session prior to start of a day, procedure, or intervention to share the plan, assign team member roles and responsibilities, establish expectations, and anticipate outcomes and likely contingencies	O	O	O	O	O
Host an ad hoc team meeting to reestablish situational awareness related to patient care, reinforce plans already in place for the delivery of care, and assess the need to adjust the plan	O	O	O	O	O
Host an informal team session designed to improve team performance and effectiveness through the review of lessons learned and reinforcement of positive behaviors after an event or procedure	O	O	O	O	O

Which of the following specific team communication tools are you aware of? Please select all that apply. If none, leave blank. *[Randomize choices]*

SBAR

Call Out

Check Back

Handoff

Brief

Huddle

Debrief

Describe the accuracy/frequency of the following statements related to openness of communication on your service or in your department. If you are unsure or a situation does not apply, please select N/A. *[Randomize choices]*

**Table:**

	*Never*	*Rarely*	*Sometimes*	*Most of the time*	*Always*	N/A
In my service/department, staff speak up if they see something that may negatively affect patient care	O	O	O	O	O	O
When staff in my service/department see someone with more authority doing something unsafe for patients, they speak up	O	O	O	O	O	O
When staff in my service/department speak up, those with more authority are open to their patient safety concerns	O	O	O	O	O	O
In my service/department, staff are afraid to ask questions when something does not seem right	O	O	O	O	O	O

Are you aware of the TeamSTEPPS team training framework?

Yes

No

Have you or your colleagues undergone formal training to enhance teamwork and communication in your hospital?

Yes

No

I'm not sure

These final questions will be used for classification purposes only.

What is your gender?

Female

Male

Nonbinary

I prefer not to answer

*[If Q3* = *Veterinarian]* How long have you been practicing veterinary medicine?

Less than 5 years

5–9 years

10–14 years

15–19 years

20 years or more

I prefer not to answer

*[If Q3<> Veterinarian]* How long have you been in the veterinary field?

Less than 5 years

5–9 years

10–14 years

15–19 years

20 years or more

I prefer not to answer

Thank you for participating in this survey. Your contribution will help enhance our understanding of teamwork and communication in specialty and emergency veterinary medicine.
